# An Optimization Model with Network Edges for Multimedia Sensors Using Artificial Intelligence of Things

**DOI:** 10.3390/s21217103

**Published:** 2021-10-26

**Authors:** Amjad Rehman, Khalid Haseeb, Tanzila Saba, Jaime Lloret, Sandra Sendra

**Affiliations:** 1 Artificial Intelligence & Data Analytics Lab (AIDA) CCIS Prince Sultan University, Riyadh 11586, Saudi Arabia; rkamjad@gmail.com (A.R.); drstanzila@gmail.com (T.S.); 2 Department of Computer Science, Islamia College Peshawar, Peshawar, Khyber Pakhtunkhwa 25000, Pakistan; khalid.haseeb@icp.edu.pk; 3 Instituto de Investigación para la Gestión Integrada de Zonas Costeras (IGIC), Universitat Politècnica de València, Camino de Vera, s/n., 46022 València, Spain; 4 School of Computing and Digital Technologies, Staffordshire University, Stoke ST4 2DE, UK

**Keywords:** multimedia sensors, optimizing resources, software-defined networks, delay controlled, artificial intelligence of things

## Abstract

In modern years, network edges have been explored by many applications to lower communication and management costs. They are also integrated with the internet of things (IoT) to achieve network design, in terms of scalability and heterogeneous services for multimedia applications. Many proposed solutions are performing a vital role in the development of robust protocols and reducing the response time for critical networks. However, most of them are not able to support the forwarding processes of high multimedia traffic under dynamic characteristics with constraint bandwidth. Moreover, they increase the rate of data loss in an uncertain environment and compromise network performance by increasing delivery delay. Therefore, this paper presents an optimization model with mobile edges for multimedia sensors using artificial intelligence of things, which aims to maintain the process of real-time data collection with low consumption of resources. Moreover, it improves the unpredictability of network communication with the integration of software-defined networks (SDN) and mobile edges. Firstly, it utilizes the artificial intelligence of things (AIoT), forming the multi-hop network and guaranteed the primary services for constraints network with stable resources management. Secondly, the SDN performs direct association with mobile edges to support the load balancing for multimedia sensors and centralized the management. Finally, multimedia traffic is heading towards applications in an unchanged form and without negotiating using the sharing of subkeys. The experimental results demonstrated its effectiveness for delivery rate by an average of 35%, processing delay by an average of 29%, network overheads by an average of 41%, packet drop ratio by an average of 39%, and packet retransmission by an average of 34% against existing solutions.

## 1. Introduction

A novel paradigm known as the internet of things (IoT) [1,2,3] emerged in the past decade due to the development of wireless technologies. This paradigm was introduced by Kevin Ashton in 1998 as a way to connect things or objects to the internet. The IoT now has many applications, such as smart homes, smart cities, transportation, healthcare, etc., supporting the community with real-time data collection and analysis [4,5,6]. Multimedia internet of things (MIoT) is promising for multimedia communication, in bringing novelties and providing an emerging model with the integration of constraint-oriented sensor networks [7,8]. The multimedia industry is comprised of graphical data, smart machines, embedded systems, and media servers that increase the efficiency of production in an optimized manner [9,10,11]. IoT-based sensors are distributed and installed in various objects for observing environment conditions and sending the collected information to the end-user over the internet. In recent decades, many applications, such as agriculture, healthcare, military, vehicles, multimedia, etc., have offered smart services to remote users as well as controllers for physical communication [12,13,14]. However, the limited resources of IoT nodes offer various restrictions for real-time applications. Therefore, most existing solutions cannot be directly applied to multimedia-based networks. Moreover, with the increasing network scale, in terms of IoT-based cameras, vehicles, sensors, etc., these physical objects must forward a huge amount of data, depleting their battery power and explicitly decreasing the network performance. Unlike a wired network, wireless topologies are more adaptive toward sharing and managing data transmission among available communication channels [15,16,17,18]. However, the wireless medium is shared, and many security threats are open for IoT-based networks. In MIoT, a huge amount of video and audio data are forwarded from sensors to the public cloud for further processing and storage purposes. However, most solutions impose additional communication overheads to maintain the multimedia routing phase. Recently, many researchers [19,20,21] have focused on coping with the routing policies in MIoT networks while considering the resource constraints of sensors. Moreover, data security cannot be avoided in the environment of the MIoT network due to the presence of malicious machines on the internet; such devices may leak data privacy and compromise the transmission system among IoT objects [22,23,24]. This work presents a multimedia internet of things model for quality assurance with the collaboration of intelligent edges and security against potential threats. It improves the development of the multimedia industry, in terms of data delivery, by incorporating intelligent edges with minimal time delays. The proposed model decreases the chances of data congestion over the wireless channels in transmitting multimedia data, with efficient utilization of a load on MIoT nodes. Moreover, the proposed model copes with vulnerable attacks from malicious entries and increases the performance of the multimedia industry by maintaining privacy and integrity. Furthermore, the security phase in the proposed model deals with unauthorized access among malicious machines and safely stores the data on the media systems for end-users.

This article presents an optimization model with SDN architecture for multimedia sensors using artificial intelligence of things to provide reliable services, in terms of QoS, and offers efficient performance for constraint resources. Moreover, the proposed model supports trustworthy data delivery to network applications without compromising the identities of devices and content. It utilizes the artificial intelligence of things with mobile edges to offer multi-hop routing services and to attain low-cost communication overhead. The initial routes are constructed using the basic requirements of any network domain with the consideration of quality factors. Moreover, mobile edges ‘perform’ as borders and control the flow management with SDN controllers. Mobile edges interact with the control plane to keep the latest information of the multimedia traffic and network status. Accordingly, the controller fetches the information from the control plane to know the exact situation of the network and helps to manage the network resources efficiently in a centralized manner. Moreover, the controller and switches utilize a low-cost secret sharing scheme to cope with information privacy and identify the uncertain multimedia traffic, increasing the efficacy of the communication system. The proposed model not only provides higher bandwidth for large size media data using mobile edges, it also protects the network data against anonymous behaviors. The three main contributions of the proposed model are as follows:

1. It offers a learning approach, with a node prediction-based multimedia algorithm by exploring the mobile edges; it attains high delivery performance with efficient management of network bandwidth.
2. It offers a low-cost computation algorithm for constraint resources, with the integration of SDN technology and boundary edges for reducing the response interval, and delays constraint multimedia applications.
3. The multimedia traffic protects against different interference attacks and centralizes the detection mechanism by increasing the support of the network applications.

The rest of the paper is as follows: Section 2 presents the related work and limitations of the existing solutions. Section 3 introduces and discusses the proposed model. In Section 4, we present the numerical analysis and results. We conclude the research work in Section 5.

## 2. Related Work

In the smart industry [25,26,27], MIoT nodes perform a vital role in collecting and distributing data to end-users. The MIoT nodes are sensors that are used to observe the reading of various physical objects in the industry and contribute to productivity. The MIoT network is connected using wireless technology, and most of the proposed solutions are prone to failure in the case of dynamic topologies. Optimizing the data routing in the multimedia-based network (without disrupting the connected users or decreasing delay time) presents significant research challenges. Moreover, security is necessary toward protecting the privacy of multimedia data, maintaining integrity against malicious attacks. The authors proposed a lightweight blockchain architecture to decentralize the authentication mechanism and claimed the effectiveness of the proposed framework for smart industrial environments. The authors of [28] proposed a novel security-by-design method for the security of the industrial internet of things (IIoT) and demonstrated its applicability by applying it to a real case study of an IIoT scenario from the maritime sector. Their security method involves analyzing the IIoT environment at two different levels—the modeling level and the simulation level. At the modeling level, the method ensures modeling and analysis of connections between IIoT components, and at the simulation levels, it provides a set of algorithms for the automatic identification of potential attack paths and categorization of the importance of such paths. The authors claimed that the proposed method helped in the identification of security mechanisms to cope with attacks on critical assets. Due to the emergence of IIoT [29], process industries have adopted wireless sensor-actuator networks (WSANs) for the accomplishment of control applications. An end-to-end communication delay in such networks can be minimized by using efficient real-time routing. The authors proposed a conflict-aware real-time routing scheme for industrial WSANs. The proposed routing scheme is evaluated on a physical WSAN test bed based on simulations and experiments that show a three-fold improvement in the real-time capacity of WSANs. Reliability and high requirements for real-time communications are very important in IIoT. The authors of [30] proposed a many-objective optimization algorithm based on the dynamic reward and penalty mechanism (MaOEA-DRP). It optimizes the shared validation validity model. Moreover, it achieves an optimized blockchain sharding method. The simulation-based experimental results are proven to have significant improvements over other solutions. The authors in [31] proposed a novel clustering method based on power demand, which assures the security of data information in IIoT-based applications using machine learning. In a first step, from mutual information of the primary channel and eavesdropping channel, the security capacity of the system is calculated. After security capacity calculation, and then keeping the constraint of the maximum transmit power, an optimal transmit power is found using the deep learning technique, which maximizes the security capacity of the system. In the final stage, the network is clustered according to the calculated power demand. In [32], the authors proposed a routing algorithm that integrates various phases, such as dynamic cluster formation and cluster head selection with multi-path routing formation. It reduces the energy consumption and routing overheads among the nodes. The proposed algorithm utilizes a genetic algorithm (GA)-based meta-heuristic optimization and dynamically chooses the best path by using the cost function. The set of experiments were conducted and analyzed, showing improved performances compared to other solutions. The authors in [33] proposed a scheme and the wireless multimedia sensor network in collecting data. Firstly, mobile sensor nodes were grouped in the cluster and a single cluster head was selected for each cluster. Secondly, the selected CHs verified the identities of the mobile sink nodes and then forwarded the multimedia data. The results showed significant performance when compared to other work. The authors in [34] proposed a resource scheduling and secure data transmission of IIoT data using SoftMax-DNN and improved RSA techniques. The authors validated and evaluated the proposed techniques and existing techniques, using simulation in JAVA and NS3 platforms, by evaluating various performance metrics in terms of latency and throughput. The proposed scheduling algorithm uses the NDRF-SSA clustering and SHA-512 algorithm; compared with existing techniques, it attains the lowest latency, the lowest energy consumption, and the highest network lifetime. In [35], a smart collaborative routing protocol with low delay and high reliability was proposed, contributing to the mixed link scenarios. The researchers constructed a one-hop delay model and analyzed the possible effects of the media access control (MAC) layer. Moreover, data forwarding, maintenance, and efficient policies were made to improve the performance of the routing protocol. Based on the experimental results, it was observed that the ratio of latency decreased compared to the existing solution. In [36], the authors proposed a mobile cloud-based scheduling strategy for the IIoT. Different computing solutions, i.e., fog, mobile, and edge computing could be combined in IIoT, allowing offloading of the execution of any task on the cloud system. The proposed solution models the problem of task scheduling to optimize the energy consumption issue. It uses genetic algorithms while taking into account task dependency, data transmission, and resource constraints. The experiments were conducted; the results showed significant improvement of the proposed solution when compared to the existing work.

The technology of the IoT and mobile edges are broadly utilized for data sensing and support efficient network structure. Such systems facilitate many network applications, i.e., healthcare, military, farming, multimedia, agriculture, etc. However, the devices and network sensors are restricted in various operations and resources. Such limitations impose many difficulties in managing the network stability and are not able to fulfill the users’ demands. Nowadays, traditional solutions are not able to support real-time data collection with a high amount of risky threats. Although some solutions are proposed in the literature, they are not fully accurate in terms of delay tolerance and delivery performance, especially when the network grows rapidly. Moreover, it was also observed that many proposals have failed to provide light cost authentication.

## 3. Proposed Optimization Model

**Figure 1:**
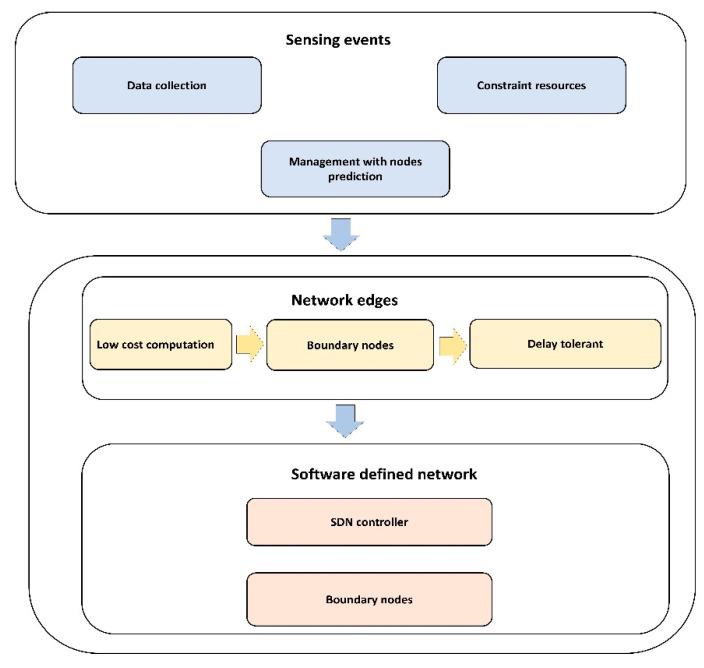
Block diagram of the proposed model.

This section presents a detailed discussion of the proposed optimization model with the integration of SDN architecture and mobile edges. It improves the efficacy of the delay constraint multimedia applications and supports the system in reacting trustworthy in case of unknown objects. Figure 1 depicts the workflow of the proposed model. It is comprised of three main blocks: (i) network sensing; (ii) network edges; and (iii) software-defined network architecture. In the first block, the sensors sense the multimedia data and achieve a QoS-aware algorithm to lower the overheads on constraint resources. The second block offers mobile edges that can collaborate with the sensing layer and SDN controller. It decreases the delay factor while routing the multimedia traffic and offers the delay-tolerant solution. In the last, SDN architecture is utilized to centralize the overall control on the network infrastructure. It not only provides better resources management, but also supports data security with the secret sharing scheme. This block increases the trust among network applications and facilitates the boundary nodes to perform lightweight data encryption with mutual authentication.

The proposed model contains three main phases. All of them operate independently and interact with each other to support the network application with an affordable load on the IoT network. The communication of the proposed model is divided into IoT sensing, network edges, and SDN levels. In the beginning, we consider various MIoT sensors to sense and transmit the multimedia traffic using mobile edges. The mobile edges are movable in a fixed radius and have high resources for processing and data storage. MIoT nodes are not able to directly interact with SDN sink node. They can transfer the multimedia data towards the sink node using the nearest mobile edges. In the proposed model, the mobile edges perform a very crucial role in decreasing the excessive response time and improving the delivery performance for delay-tolerant applications. MIoT nodes, before initiating data forwarding, share their statistics, establish forwarding tables to retain up-to-date information in their proximity, and train themselves for optimum outcomes. The information comprises of identities ID, residual energy, distance, and link disturbing. Afterward, they perform an authentication phase to verify with each other, using the certificate tokens. All of the nodes exchange the certified tokens that are signed by the master key $k_{m}$ of each node. Upon successful reception of the token, each node marks the entities to their forwarding tables. Moreover, each node forwards its tokens to the nearest edge nodes, so their association can be created in the upper layer. Mobiles edges advertise their identities IDs and positioning coordinates on a regular interval, so the nearest nodes could detect their latest positions and update the forwarding tables. Moreover, the mobile edges transmit their local tables to SDN controllers using deployed switches and routers. In this way, the control plane gets the updated information of the network layer and manages the resources efficiently for the constraint-oriented devices.

Afterward, the proposed model adjusts the data flow among MIoT nodes using intelligent decisions. The source advertises the route request RREQ packet to identify the initiate route for data routing. Upon receiving, the neighbor nodes respond with status information $n_{s}$ along with their identities $ID_{i}$. The status information is determined by utilizing the distance $D_{t}$, received signal strength indicator RSSI, and re-transmission interval $Rt_{int}$ factors, as given in Equation (1).

(1) $$n_{s}=min(D_{t}+Rt_{int})+max\left(RSSI\right),$$

In Equation (2), $D_{t}$ is the integration of a two-level distance dist, i.e., the Euclidean distance, from the source node to neighbor α and from neighbor α to mobile edge β, as given below.

(2) $$D_{t}=dist(\alpha ,\;\beta ),$$

Using the computed value of $n_{s}$, the source node updates its forwarding table and sends the data toward the mobile edge based on the hop-to-hop paradigm. However, it might be a case where the mobile edge is directly accessible by the source node. In such a situation, the source node sends the data directly without evaluation $n_{s}$ values. In the proposed model, the network edges are mobile and adjust the coordinates frequently; thus, its latest position $e_{ps}$ can be determined using Equation (3).

(3) $$e_{ps}=(P_{0}-P_{i})/S$$

In Equation (3), $P_{i}$ and $P_{0}$ are initial and current 2D coordinates, whereas S is the speed of the mobile edges. The positioning coordinates are obtained using the installed global positioning system (GPS) on mobile edges. Later, the network edges on different levels initiate their work collaboratively, to deliver the MIoT data toward the SDN controller using employed switches and routers.

In the proposed model, SDN deploys on the top level and it supervises all of the network operations in a centralized manner. To support the data protection on each level, SDN generates a secret key S, which is to be divided between the set of n network edges based on Shamir’s secret sharing scheme [37]. It is also called (t,n) threshold based secret sharing, such that, any t subset of network edges are sufficient to recreate the secret key S. However, less than t or a fewer number of subkeys cannot reconstruct the secret key S. Afterwards, the SDN controller transmits the share of the key $S_{i}$ to the network edges, which is also digitally signed by the SDN master key $mk_{SDN}$. On receiving the secret share, each network edge node is first verified by decrypting the secret share, and afterward, it further floods towards an individual node that is associated with the network edge. Let us consider that $m_{i}$ denotes message pieces that must be sent from the nodes toward the network edges. Then, nodes perform a mapping function using a set of subkeys ($S_{0},\;S_{1},\ldots ,S_{t-1}$), and are digitally signed by their master keys $mk_{n}$, as given in Equation (4).

(4) $$E_{i}=mk_{n}(\left(S_{i},\;m_{i}\right),xor)\;$$

After receiving the encrypted data $E_{i}$, the network edges verify it, and upon successful verification, the data are transmitted toward the controller using deployed switches, as given in Equation (5).

(5) $$X_{i}=f(IV+\;(m_{i},S_{i})\;xor)+D$$

where IV is a nonce, and is used to make the encryption process more randomized, and D denotes the digital signature of the network edge. When the SDN controller receives the encrypted data $X_{i}$, it performs a decryption function $Y_{i}$, as given in Equation (6), and forwards to the application devices for connected nodes that can retrieve it for the needed purpose.

(6) $$Y_{i}=((m_{i},X_{i})\;xor)\;$$

**Figure 2:**
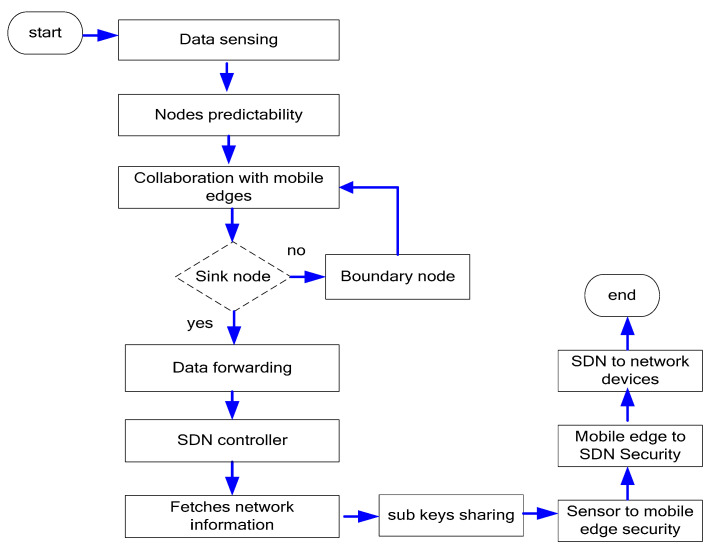
Flow chart of the proposed model.

Figure 2 illustrates the flow chart of the proposed model. It initiates a network-sensing component using IoT devices and multimedia sensors. The sensors are very restricted for resources, and cannot transmit a huge amount of media traffic; thus, the proposed model offers a QoS-aware routing algorithm, while considering the node statistics and user demands. In addition, mobile edges are utilized in the proposed model to overcome the delay factor, reducing the size of forwarding tables for sensors nodes. The mobile edges perform an interaction with both IoT nodes and the SDN controller by deployed switches and routers. The SDN controller decouples the control plane and data plane and fetches the store information from the control plane to manage the multimedia traffic with efficient data distribution and resource supervision. Moreover, the controller utilizes a threshold-based secret sharing scheme to increase the secrecy level among low level and boundary nodes. Such components support trustworthy communication from sensing nodes to network applications. The boundary nodes that perform a vital role while maintaining the node records are also securing from malicious threats, based on the SDN architecture.

## 4. Performance Evaluation

In this section, we present the simulation environment and experiments discussion. The experiments were conducted in OMNET++ [38,39], which is widely used by the research community to simulate network technologies and standards. We ran the simulations on a laptop with a 16 M cache, 256 GB RAM, and a 4.40 GHz Intel processor. The performance was evaluated against existing schemes over a 1000 × 1000 m area. We considered the transmission range of each node to be 10 m. To evaluate the security significance, we deployed 10 malicious nodes randomly. Moreover, switches and routers were deployed with the POX controller. Initially, the energy resource of each sensor node was set to 2 j. We executed the simulation for 2000 s. The size of the data block was fixed to 32 bits. The experiments were conducted based on the delivery rate, network overhead, processing delay, and packet loss rate under varying network nodes and data receiving rates. The proposed model was compared with existing solutions, i.e., MaOEA-DRP and smart collaborative routing protocol, as explained in [30,35]. The default parameters are listed in Table 1.

**Figure 3:**
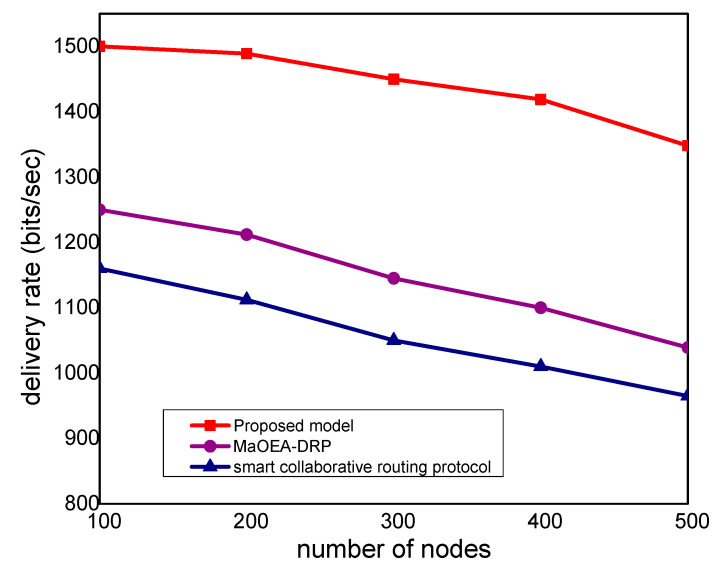
Delivery rate with the number of nodes.

**Figure 4:**
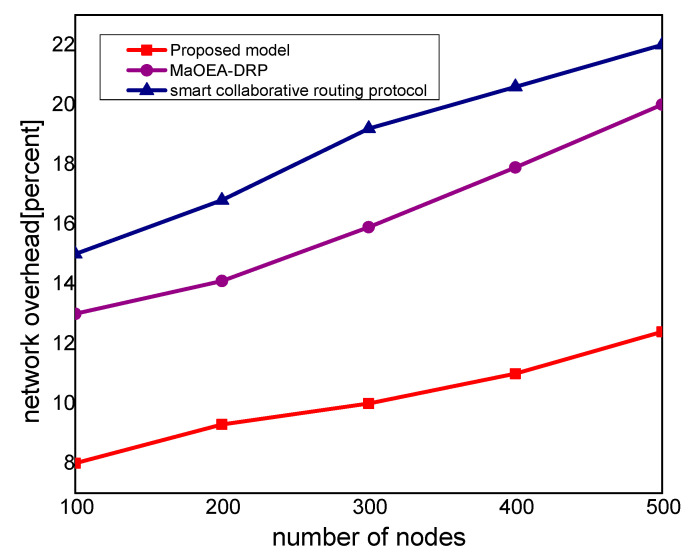
Network overhead with the number of nodes.

**Figure 5:**
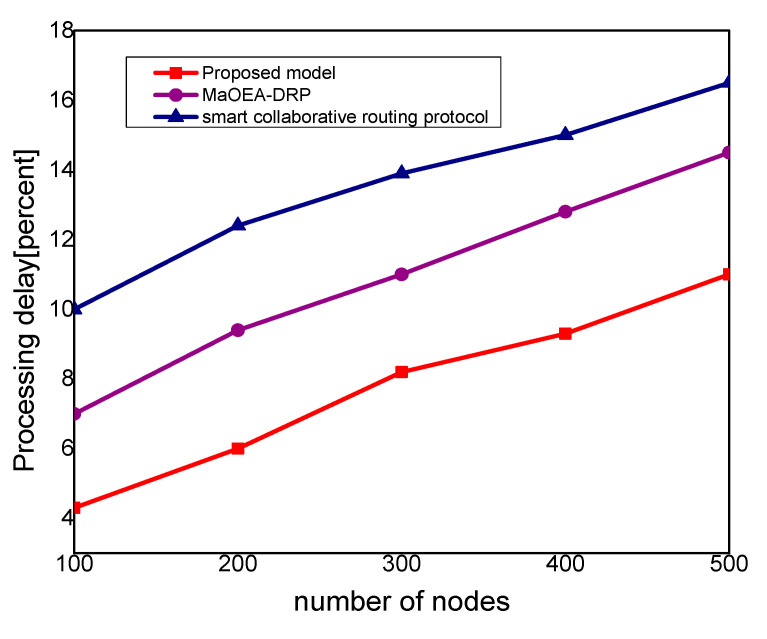
Processing delay with the number of nodes.

**Figure 6:**
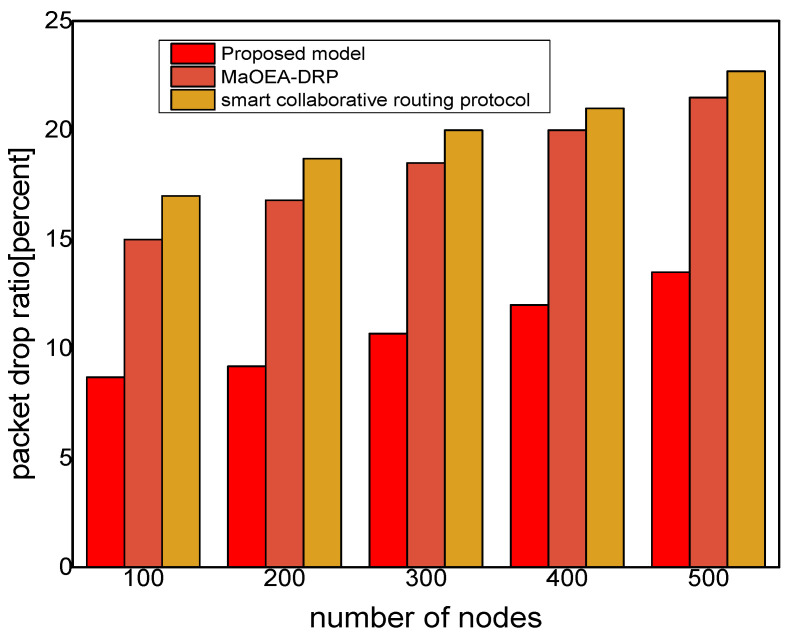
Packet drop ratio with the number of nodes.

**Figure 7:**
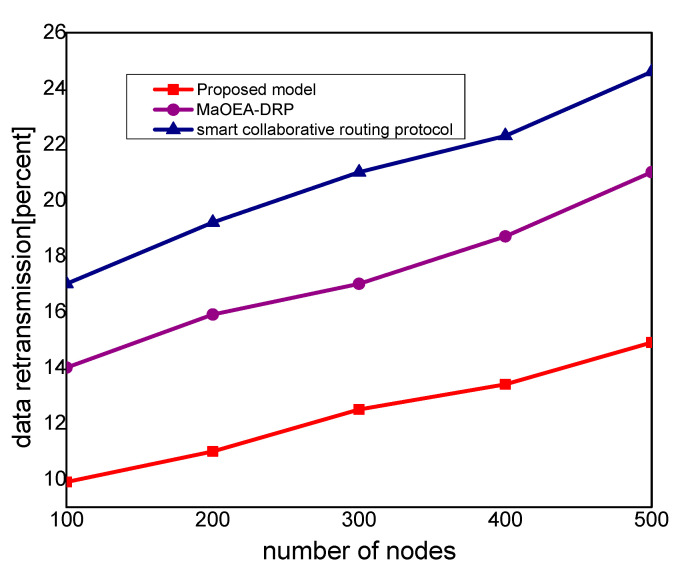
Data retransmission with the number of nodes.

In Figure 3, the experimental results illustrate that the proposed model improves the packet delivery rate by 30% and 40%, as compared to other solutions. Thus, the proposed model utilizes a multi-hop transmission system for routing the MIoT data and optimizes the forwarding decision. The intelligent decision determines the packet variability factor for the neighbor nodes and increases the packet delivery performance. Furthermore, the communication medium is protected in the existence of malicious packets and efficiently utilizes resource management. Accordingly, the proposed model minimizes the ratio of congestion on the wireless channels and achieves robust transmission. The proposed model makes use of mobile edge, computing high-performance nodes for collecting the MIoT data from sensors, and increases the efficacy of data management. Unlike other solutions, the proposed model decreases the exchange of control messages among the nodes and ultimately improves the throughput of the MIoT network. Figure 4 illustrates the performance of the proposed model for routing overheads in the comparison of existing solutions. It is noticed that the proposed model reduces the routing overheads by 38% and 44%, respectively, under a varying number of nodes. Unlike the existing solution that frequently exchanges control and route request messages among nodes in case of a larger network size, the proposed model explicitly avoids such practice. It only selects the MIoT node as a data forwarder when the selection criteria are less than a certain threshold. Moreover, the proposed model efficiently utilizes the energy resource of the MIoT nodes and decreases the rapidly routing messages. Moreover, due to the mobile edge computing nodes, the MIoT nodes enforce the least communication costs in forwarding and choosing the optimal route. Accordingly, the proposed model imposes fewer overheads on the part of MIoT nodes and improves the network performance by selecting the more reliable routes. Figure 5 demonstrates the performance results, in terms of processing time for the proposed model against other solutions. Based on the experiments, it is seen that the proposed model improves the time delay by 24% and 33% than the exiting work. The improvement is due to balancing the energy and data-forwarding load among the MIoT nodes using the packet variability factor. Moreover, using secret sharing with mobile edges, the proposed model prevents the malicious nodes from being part of the MIoT network, and avoids frequent transmission of false or bogus data packets. The proposed model efficiently utilizes the transmission power of mobile edge computing nodes and improves routing management. Using a multi-hop communication system, the proposed model decreases the chance for the selection of the longer route. Moreover, based on the packet variability factor among MIoT neighbors, the proposed model forwards the observing data timely, and with more consistency in the chosen route. Therefore, it prolongs the lifetime of the active routes and, accordingly, improves the performance of end-to-end delay remarkably. In Figure 6, the experimental results illustrate the performance of the proposed model, in terms of packet drop ratio, compared to the existing solution. It is observed that the proposed model decreases the packet drop ratio by 37% and 41%, respectively; this is due to the determination of packet variability by utilizing the distance and *RSSI* factor. It optimizes the MIoT route among constraint resources and provides strengthened peer nodes for accomplishment to data storage on the public cloud. Moreover, the proposed model offers a more secure and authenticated routing mechanism, using a secured approach that increases the confidence ratio among nodes, and incurs minor data lost in the occurrence of malicious entities. In Figure 7, the proposed model evaluates the reliability of the proposed model in the comparison with an existing solution. It is seen from the simulation-based results under a varying number of nodes that the proposed model improves the packet drop ratio against other work by 29% and 39%. It is due to the incorporation of the reliable routing and secure cryptosystem for the MIoT network, improving productivity for the industry. The use of mobile edge nodes also decreases the energy consumption among MIoT nodes and, ultimately, the lifetime of the network increases with the efficient practice of data aggregation/fusion. Moreover, the MIoT data are securely transmitted to the cloud for further processing and storage based on the lightweight cryptography asymmetric algorithm, which increases the reliability among nodes against network threats.

## 5. Conclusions

In this paper, the optimization model with mobile edges for multimedia sensors using artificial intelligence of things is presented, which aims to increase the management of network resources in multimedia traffic with securing transmission. It provides the real-time paradigm for critical MIoT-based applications and facilitates production with high reliability. Moreover, communication is secured under the occurrence of malicious nodes with the lightweight nodes’ power of the MIoT network using intelligent SDN technology. It gives intelligent decisions among mobile edges by evaluating the QoS features and strengthens the network performance. Moreover, nodes are authenticated with each other and secret shares by using the Shamir secret sharing scheme. The set of experiments were performed in the OMNET++ simulator, and based on the results analysis, it is proven that the proposed model remarkably increases the performance for the delivery rate, time delay, routing overheads, packet drop ratio, and reliability, than benchmark solutions. The proposed model gives some intelligence using edge computing; however, it faces some communication expenses in determining the optimal forwarders. Thus, in the future, we aim to utilize the transfer learning technique and train the IoT network with a real-time data set.

## Figures and Tables

**Table 1:** Default parameters.

Parameters	Values
Simulation area	1000 × 1000 m
Sensor nodes	100–500
Malicious nodes	10
Data block, k	32 bits
Initial energy	2 j
Transmission power	5 m
Simulation interval	2000 s
Transmission radius	5 m
Data flow	Periodic

## Data Availability

All Data is available in the manuscript.
